# MALAT1 Mediates *α*-Synuclein Expression through miR-23b-3p to Induce Autophagic Impairment and the Inflammatory Response in Microglia to Promote Apoptosis in Dopaminergic Neuronal Cells

**DOI:** 10.1155/2023/4477492

**Published:** 2023-04-06

**Authors:** Xin Geng, Yanghong Zou, Shipeng Li, Renli Qi, Hualin Yu, Jinghui Li

**Affiliations:** The Second Department of Neurosurgery, The First Affiliated Hospital of Kunming Medical University, Kunming, 650032 Yunnan, China

## Abstract

**Background:**

Parkinson's disease (PD) is a very common neurodegenerative disease that adversely affects the physical and mental health of many patients, but there is currently no effective treatment.

**Objective:**

To this end, this study focused on investigating the potential mechanisms leading to dopaminergic neuronal apoptosis in PD.

**Methods:**

Rotenone induces damage in dopaminergic neuronal MN9D cells. Apoptosis was detected by flow cytometry, and the expression of apoptosis-related proteins was detected by western blot. RT-qPCR was used to detect the expression of MALAT1 and miR-23b-3p. The expression of *α*-synuclein was detected by ELISA. A dual luciferase gene reporter assay was used to determine the targeted regulatory relationship between MALAT1 and miR-23b-3p and miR-23b-3p and *α*-synuclein. MN9D supernatant was cocultured with BV-2 cells, or BV-2 cells were treated with exogenous *α*-synuclein and then treated with an autophagy inhibitor (3-MA) and autophagy activator (RAPA). The expression of *α*-synuclein in BV-2 cells was detected by immunofluorescence. The expression of MIP-1*α*, a marker of microglial activation, was detected by ELISA. The nuclear translocation of NF-*κ*B p65 was detected by immunofluorescence. The expression of proinflammatory cytokines was detected by ELISA. Western blotting was used to detect the expression of autophagy-related proteins. Apoptosis of MN9D cells was detected after coculture of BV-2 supernatant with MN9D.

**Results:**

The expression of MALAT1 and *α*-synuclein was upregulated, while the expression of miR-23b-3p was downregulated in damaged MN9D cells, resulting in cell apoptosis. MALAT1 can negatively regulate the expression of miR-23b-3p, while miR-23b-3p negatively regulates the expression of *α*-synuclein. *α*-synuclein can enter BV-2 cells through cell phagocytosis. Coculture of BV-2 cells with *α*-synuclein or with MN9D supernatant overexpressing MALAT1 resulted in a decrease in the autophagy level of BV-2 cells and an inflammatory reaction. However, miR-23b-3p mimics and knockdown of *α*-synuclein reversed the effect of MALAT1 on autophagy and the inflammatory response of BV-2 cells. In addition, after coculture of BV-2 cells with *α*-synuclein, the level of autophagy further decreased when 3-MA was added, while the opposite result occurred when RAPA was added. After coculture of *α*-synuclein-treated BV-2 cell supernatant with MN9D cells, autophagy-impaired BV-2 promoted the apoptosis of MN9D cells, and 3-MA aggravated the autophagy disorder of BV-2 and further promoted the apoptosis of MN9D cells, while RAPA reversed the autophagy disorder of BV-2 and alleviated the apoptosis of MN9D cells.

**Conclusion:**

MALAT1 can promote *α*-synuclein expression by regulating miR-23b-3p, thereby inducing microglial autophagy disorder and an inflammatory response leading to apoptosis of dopaminergic neurons. This newly discovered molecular mechanism may provide a potential target for the treatment of PD.

## 1. Introduction

Parkinson's disease (PD) is a well-known neurodegenerative disease characterized by the progressive loss of dopamine neurons, resulting in physical impairment. PD mostly affects elderly individuals, with a prevalence of approximately 5.5% among people over the age of 60 [1]. Once suffering from Parkinson's disease, the patient's body will gradually stiffen and tremble involuntarily. In short, the patient's mobility will continue to decrease until they lose their ability to care for themselves. The main cause of PD is the massive degenerative death of dopaminergic (DA) neurons due to inclusions of aggregated alpha-synuclein (*α*-Syn) [2, 3]. The cause of neuronal death is activated microglia, which are key cells that regulate immune responses to maintain brain homeostasis and normally exhibit a neurospecific phenotype. In addition, microglia produce a variety of proinflammatory factors, including interleukin-1*β* (IL-1*β*), in the human brain [4–6], and these proinflammatory factors contribute to the development of neuroinflammation. However, neuroinflammation is often thought to trigger neurodegenerative diseases [7]. Therefore, microglia have unique regulatory functions in the central nervous system (CNS). However, factors affecting the activation of these microglia may impair their neuroprotective effects during neurodegenerative diseases, which exacerbate neuroinflammation when dopaminergic neurons are lost, and these mechanisms will serve as the basis for our study of the pathological process of PD.

It is well known that noncoding small RNAs include a variety of RNA molecules, including miRNAs and siRNAs. Among them, miRNAs are 18-23 nucleotides in length. They can silence or degrade mRNA and thus affect multiple processes, including cell death [8–10]. Reviewing a large number of studies and the clinical evidence, we know that lncRNAs are highly expressed in the brain, and researchers believe they have multiple neuromodulatory functions. Numerous studies have also shown that lncRNAs in the brains of patients with Parkinson's disease present a clear upregulation of expression trend, suggesting that they may have unique regulatory functions in neurological diseases [11].

Among them, metastasis-associated lung adenocarcinoma transcript 1 (MALAT1 or NEAT2) is a member of the lncRNA family. Numerous experimental data suggest that tumors [12] and disordered neurons [13] are sites of MALAT1 overexpression. It has been demonstrated that MALAT1 is involved in the formation of structures such as synapses [14] and it has a pivotal regulatory role in MPTP-induced PD [15].

MicroRNAs are a class of powerful small RNAs that have been discovered and studied in recent years. They can silence or degrade mRNA and thus affect many processes, including cell death [16]. Circulating miRNAs have been shown to be potential biomarkers for the diagnosis of PD [17–19]. One study has demonstrated that circulating miRNAs in plasma, serum, or serum exosomes are potential biomarkers for PD diagnosis. Moreover, it has been found that *α*-synuclein and miRNA are overexpressed in PD, AD, and dementia [20]. miR-23b-3p can directly target and regulate the expression of *α*-synuclein, leading to the development of PD [21].

Alpha-synuclein is a protein that is highly enriched in presynaptic nerve terminals. It is a soluble protein expressed in the presynaptic membrane of the CNS as a major component of the Lewy bodies, and its physiological functions include involvement in central nervous system (CNS) synaptic development, mediating DA synthesis and release, presynaptic vesicle transport, lipid metabolism, and molecular chaperone function. *α*-syn is inextricably linked with the pathogenesis of PD [21]. According to several studies, *α*-syn can be propagated between neurons [22, 23]. Areas of the brain affected by the Lewy bodies can have deleterious effects on adjacent areas, causing these areas to also form Lewy bodies. Furthermore, some experimental studies indicated that higher *α*-syn levels were associated with greater neuroinflammation in the brain, especially in the nigrostriatal pathway, increasing the effect of dopaminergic neurons on *α*-syn-induced inflammation [24]. Thus, *α*-synuclein may be a key molecule contributing to the development of PD.

It has been shown that the classical antibiotic rifampicin can prevent rotenone-induced microglial inflammation mediated by enhanced autophagy [25]. Autophagy is an important mechanism for maintaining homeostasis [26]. A review of the literature reveals that autophagy has a unique and potent function in immunosuppressive inflammatory responses [27–32]. Loss of autophagy, including *α*-synuclein degradation and microglial activation-induced neuroinflammation, has been reported to be inextricably linked to dysfunction in PD [33–37]. Factors affecting microglial activation may exhibit neuroprotective effects in neurodegenerative diseases when microglial autophagy is impaired, leading to neurodegeneration in mice [38–40], and the autophagy pathway loses its function in the process [41].

To our knowledge, PD is associated with MALAT1, miR-23b-3p, and *α*-synuclein molecules, but the exact mechanism of how they cause PD remains to be proven. Previously, it was reported that microRNA-124 regulates p62/p38 expression and promotes autophagy [42]. In addition, lncRNA SNHG14 regulates the miR-133b/*α*-Syn pathway to alleviate dopamine neuronal damage in PD [43]. Therefore, we analyzed the targeting relationship between *α*-Syn and miR-23b-3p and MALAT1 and miR-23b-3p by bioinformatics. Our objective was to investigate whether lncRNA MALAT1 acts as a sponge for miR-23b-3p to regulate *α*-synuclein expression, which affects autophagy and the inflammatory response of microglia and promotes apoptosis of dopamine neural cells. The ultimate goal was to provide a scientific basis for the prevention and treatment of neurodegenerative diseases.

## 2. Materials and Methods

### 2.1. Cells and Transfection

MN9D (BFN60808672) and BV-2 (BFN608006363) cells were purchased from the Cell Bank of the Chinese Academy of Sciences (BFB, Qingqi (Shanghai) Biotechnology Development Co.), and they were grown and passaged in DMEM (ExCell Bio, China) containing 10% fetal bovine serum. MALAT1, miR-23b-3p mimic, and sh-*α*-synuclein were transfected into MN9D cells with Lipofectamine 3000 transfection reagent (Invitrogen, Carlsbad, CA, USA), and the supernatant was collected and cocultured with small neuroglial cells.

### 2.2. Detection of Apoptosis by Flow Cytometry

An Annexin V-FITC/PI kit (BD Pharmingen) was used as directed by the manufacturer, and then, Annexin V and PI fluorescence were determined at the reference emission wavelengths by a Beckman cytometer (BD Biosciences) and FlowJo software (Beckman Coulter, TE, CytoFLEX).

### 2.3. RT-qPCR

A TRIzol RNA extraction kit (Invitrogen, Carlsbad, CA, USA) was used to extract total RNA from the collected cells. Then, the first strand of cDNA was synthesized using the total RNA of the sample as a template, and the cDNA obtained was used as a template for qPCR amplification. The subsequent PCR process was completed using cDNA and U6 as the internal reference. A SYBR Green qPCR kit (TaKaRa, Dalian, China) was used according to the manufacturer's instructions. Then, the expression levels in the cells and tissues were calculated using the 2^–*ΔΔ*Ct^ method. The primer sequences are displayed in Table 1.

### 2.4. Western Blotting Assay

The total protein of each group was extracted, and the protein concentration was determined. Then, the samples were subjected to electrophoresis separation and transferred to a membrane. Then, the membrane was incubated with primary antibodies (Abcam, UK), including anti-Bcl-2 (ab32124, 1 : 1000), anti-Bax (ab32503, 1 : 2000), anti-Caspase-3 (ab32042, 1 : 500), anti-*α*-synuclein (ab138501 1 : 2000), anti-LC3 (ab192890, 1 : 2000), anti-Beclin 1 (ab207612, 1 : 5000), anti-p62 (Abcam, ab207305, 1 : 1000), and a goat anti-rabbit secondary antibody (Abcam, ab205718, 1 : 2000). After incubation, the membranes were assessed for expression semiquantitatively by enhanced chemiluminescence (ECL) chromogenic and gel imaging. The experiment was repeated three times.

### 2.5. Enzyme-Linked Immunosorbent Assay (ELISA)

Samples and standards were added to 96-well plates coated with the relevant antibodies using ELISA kits (Solarbio, MIP-1*α*, TNF-*α*, IL-1*β*, and IL-6; Beyoncé Biotechnology, *α*-synuclein and INF-*γ*), and the plates were washed 5 times. Biotinylated antibody was added and incubated for 1 h at 37°C. Then, the enzyme conjugate working solution was added and incubated in the dark. Chromogenic substrate was added and incubated at 37°C for 15 min. Finally, the absorbance value was measured and compared to an enzyme standard, and the sample concentration was calculated according to the standard curve.

### 2.6. Target Binding Site Prediction and Dual Luciferase Gene Reporter Assay

StarBase predicted targeted binding sites for MALAT1 and miR-23b-3p and targeted binding sites for miR-23b-3p and *α*-synuclein. The miR-23b-3p and MALAT1 or *α*-synuclein 3′-UTR binding site and its mutated sequence were inserted into the pmirGLO dual luciferase vector (GenePharma, Shanghai, China). The constructed vector was cotransfected with miR-23b-3p mimic and its negative control (NC mimic) into cells using Lipofectamine 2000. Forty-eight hours after transfection, luciferase activity was detected using a dual luciferase reporter assay kit (Hanbio Biotechnology, Shanghai, China).

### 2.7. Immunofluorescence

Microglial BV-2 cells were cocultured with PBS, rotenone-treated MN9D cell supernatant, rotenone-treated MN9D cell supernatant + cytochalasin D (CCD), *α*-synuclein, and *α*-synuclein + CCD, and the cultured cells were fixed and permeabilized and then incubated with Cy3 fluorescently labeled anti-*α*-synuclein (Abcam, ab138501, 1 : 150) antibody, as well as goat anti-rabbit IgG secondary antibody (Abcam, AlexaFluor®555, 1 : 200) under different conditions. Finally, after Hoechst 33342 (1 : 200; Sigma-Aldrich) staining, images were taken by laser scanning confocal microscopy using a fluorescence microscope (Nikon, Tokyo, Japan). Moreover, the percentage of positive cells was calculated by the ImageJ blinded method.

### 2.8. Detection of NF-*κ*B p65 Nuclear Translocation

Cells were fixed according to the instructions of the Cellular NF-*κ*B Translocation Kit (Beyotime Biotech) and then incubated for 1 h with closure buffer. After washing, the cells were incubated with anti-NF-*κ*B p65 antibody (Abcam, ab183559, 1 : 100) for 1 h at room temperature, followed by Cy3 fluorescently labeled goat anti-rabbit IgG (Abcam, ab150077, 1 : 1000) secondary antibody. Afterward, the coverslips were incubated with phosphatidylinositol 3-kinase (DAPI) for 5 min. Finally, images were taken with laser scanning confocal microscopy (LSCM) (Zeiss, Thornwood, NY).

### 2.9. Statistical Analysis

Prism 7.0 software (GraphPad) was used to analyze the data. Data are given as mean ± standard deviation (SD). In the statistical comparison, the data that conformed to the normal distribution were analyzed by Student's *t*-test for comparisons between two groups, one-way ANOVA was used for comparisons between multiple groups, and a nonparametric test was used for the data that did not conform to a normal distribution. *P* < 0.05 indicates statistical significance.

## 3. Results

### 3.1. Differential Expression of MALAT1, miR-23b-3p, and *α*-Synuclein in Damaged Dopaminergic Neuronal Cells

**Figure 1:**
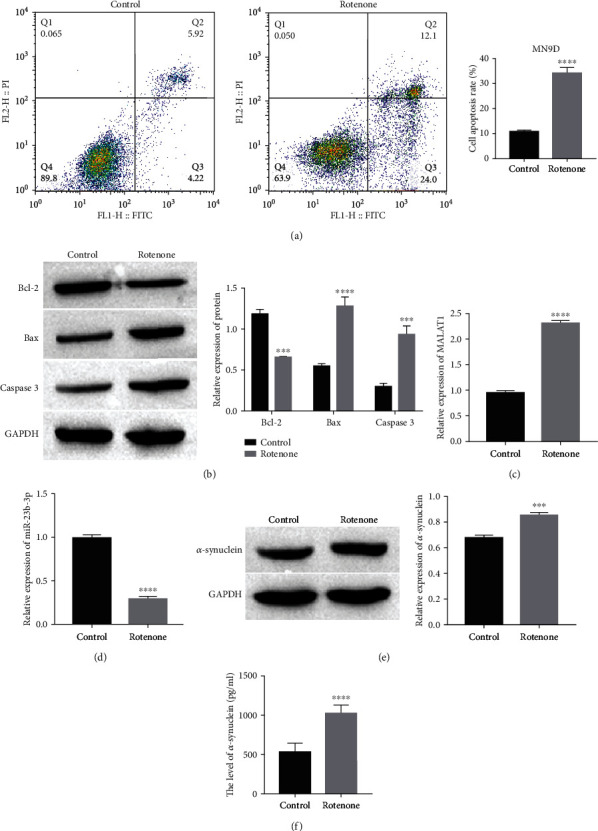
Differential expression of MALAT1, miR-23b-3p, and *α*-synuclein in damaged dopaminergic neuronal cells. (a) Apoptosis of dopaminergic neurons in MN9D cells was detected by flow cytometry, ^∗∗∗∗^*P* < 0.0001 vs. control. (b) The expressions of Bcl-2, Bax, and Caspase 3 were detected by western blot, ^∗∗∗^*P* < 0.001 and ^∗∗∗∗^*P* < 0.0001 vs. control. (c, d) The expressions of MALAT1 and miR-23b-3p were detected by RT-qPCR, ^∗∗∗∗^*P* < 0.0001 vs. control. (e) The protein expression of *α*-synuclein was detected by western blot, ^∗∗∗^*P* < 0.001 vs. control. (f) The level of *α*-synuclein was detected by ELISA, ^∗∗∗∗^*P* < 0.0001 vs. control.

From the results of flow cytometry, we found that the apoptosis rate of MN9D cells showed an upward trend in the rotenone group (Figure 1(a)). Moreover, after observing the western blot results, we found that the expression of the antiapoptotic protein Bcl-2 was downregulated, but the proapoptotic proteins Bax and Caspase 3 showed the opposite trend in the rotenone group (Figure 1(b)). After evaluation by RT–qPCR, the quantitative data showed that the expression of MALAT1 was upregulated, but miR-23b-3p showed the opposite trend in the rotenone group (Figures 1(c) and 1(d)). Moreover, by analyzing the data detected by western blot, we concluded that rotenone promoted the expression of *α*-synuclein in MN9D cells (Figure 1(e)), while ELISA also revealed that the expression of *α*-synuclein in the MN9D cell supernatant was also upregulated after rotenone treatment (Figure 1(f)). These results suggest that rotenone can damage dopaminergic neuronal cells and promote the apoptosis of injured cells. In addition, the differential expression of MALAT1 and miR-23b-3p, together with *α*-synuclein, is inextricably linked to dopaminergic neuronal cell damage and that large amounts of *α*-synuclein protein accumulate when dopaminergic neuronal cells are injured.

### 3.2. MALAT1 Acts as a miR-23b-3p Sponge to Regulate *α*-Synuclein Expression

**Figure 2:**
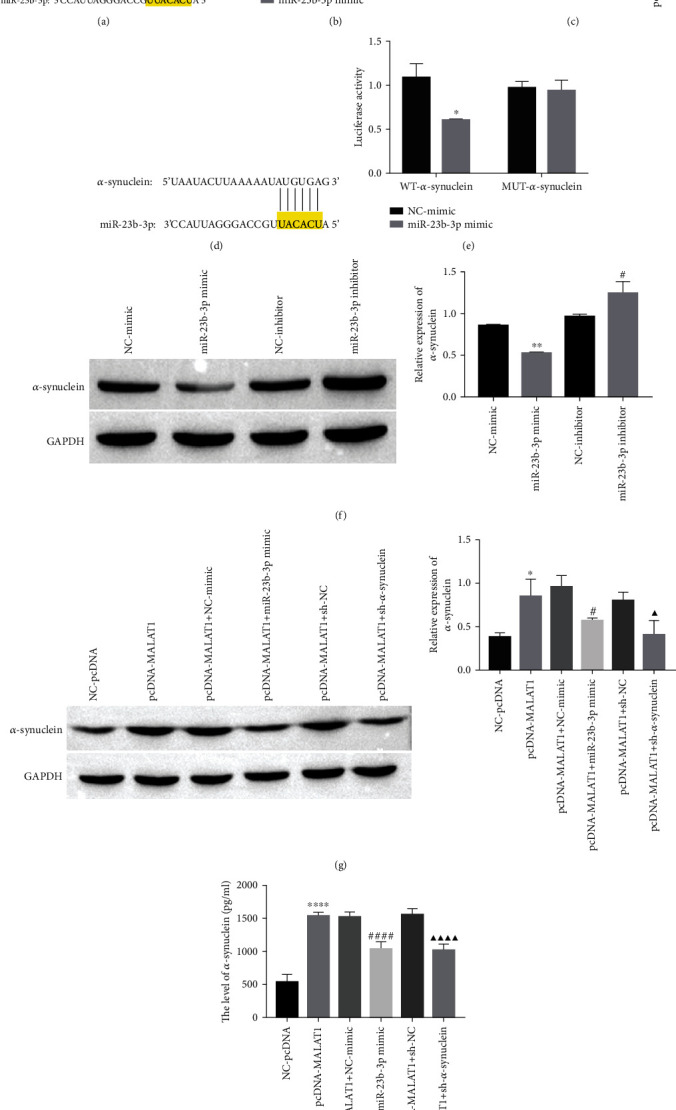
MALAT1 acts as a miR-23b-3p sponge to regulate *α*-synuclein expression (MN9D cells). (a) StarBase predicts the targeting binding site between MALAT1 and miR-23b-3p. (b) Dual luciferase reporter assay was used to verify the targeting binding relationship (MALAT1 and miR-23b-3p), ^∗∗^*P* < 0.01 vs. NC-mimic. (c) The expression of miR-23b-3p was detected by RT-qPCR, ^∗∗∗∗^*P* < 0.0001 vs. si-NC and ^#^*P* < 0.05 vs. pcDNA. (d) StarBase predicts the targeting binding site between miR-23b-3p and *α*-synuclein. (e) Dual luciferase reporter assay was used to verify the targeting binding relationship (miR-23b-3p and *α*-synuclein), ^∗^*P* < 0.05 vs. NC-mimic. (f) The expression of *α*-synuclein was detected by western blot, ^∗∗^*P* < 0.01 vs. NC-mimic and ^#^*P* < 0.05 vs. NC-inhibitor. (g) The expression of *α*-synuclein was detected by western blot, ^∗^*P* < 0.05 vs. NC-pcDNA, ^#^*P* < 0.05 vs. pcDNA-MALAT1+NC-mimic, and ^▲^*P* < 0.05 vs. pcDNA-MALAT1+sh-NC. (h) The level of *α*-synuclein was detected by ELISA, ^∗∗∗∗^*P* < 0.0001 vs. NC-pcDNA, ^####^*P* < 0.0001 vs. pcDNA-MALAT1+NC-mimic, and ^▲▲▲▲^*P* < 0.0001 vs. pcDNA-MALAT1+sh-NC.

The bioinformatics website StarBase was used to predict the targeting relationship. miR-23b-3p targets MALAT1 based on the results predicted by StarBase and the dual luciferase gene detection experiment (Figures 2(a) and 2(b)), and the RT-qPCR assay results further identified that MALAT1 targets and negatively regulates miR-23b-3p (Figure 2(c)). Similarly, StarBase predicted that miR-23b-3p has a target binding site for *α*-synuclein (Figure 2(d)). A dual luciferase gene reporter assay verified the binding relationship between *α*-synuclein and miR-23b-3p (Figure 2(e)), and western blot analysis identified that miR-23b-3p targets and negatively regulates *α*-synuclein expression (Figure 2(f)).

To further understand their regulatory role, in MN9D cells, miR-23b-3p mimics were transfected after overexpression of MALAT1 or *α*-synuclein was knocked down after overexpression of MALAT1, and western blot and ELISA were used to detect the expression of *α*-synuclein under different treatments. After observing the western blot results, we found that the expression of *α*-synuclein showed an upward trend in the pcDNA-MALAT1 group compared with the NC-pcDNA group, and transfection of the miR-23b-3p mimic effectively reversed the promotion of *α*-synuclein expression by MALAT1 (Figure 2(g)). Similarly, knocking down the expression of *α*-synuclein (sh-*α*-synuclein) effectively reversed the promotion of *α*-synuclein expression by MALAT1, i.e., compared with the pcDNA-MALAT1+sh-NC group, the pcDNA-MALAT1+sh-*α*-synuclein group exhibited downregulated *α*-synuclein expression (Figure 2(g)). ELISA also showed that MALAT1 could increase the expression of *α*-synuclein in the supernatant of MN9D cells, while transfection of miR-23b-3p mimic or knockdown of *α*-synuclein could effectively reverse the effect of MALAT1 on *α*-synuclein expression promotion and it reduced *α*-synuclein expression in MN9D cell supernatants (Figure 2(h)). The above results suggest that MALAT1 can regulate *α*-synuclein expression by affecting miR-23b-3p.

### 3.3. *α*-Synuclein Enters Microglia through Cytophagocytosis

Previous findings showed that *α*-Syn can be transmitted between cells [22]. To investigate the role of *α*-synuclein in microglia, we treated microglia (BV-2) with PBS as a control, extracted the supernatant of rotenone-treated MN9D cells and cocultured it with BV-2 cells as the supernatant group, and then added CCD (phagocytosis inhibitor cytochalasin D) as the supernatant +CCD group. Meanwhile, *α*-synuclein was added exogenously to BV-2 cells as the *α*-synuclein group, and *α*-synuclein+CCD grouping was also set up after the addition of *α*-synuclein and then the phagocytosis inhibitor cytochalasin D to determine the phagocytosis of *α*-synuclein by small glial cells.

**Figure 3:**
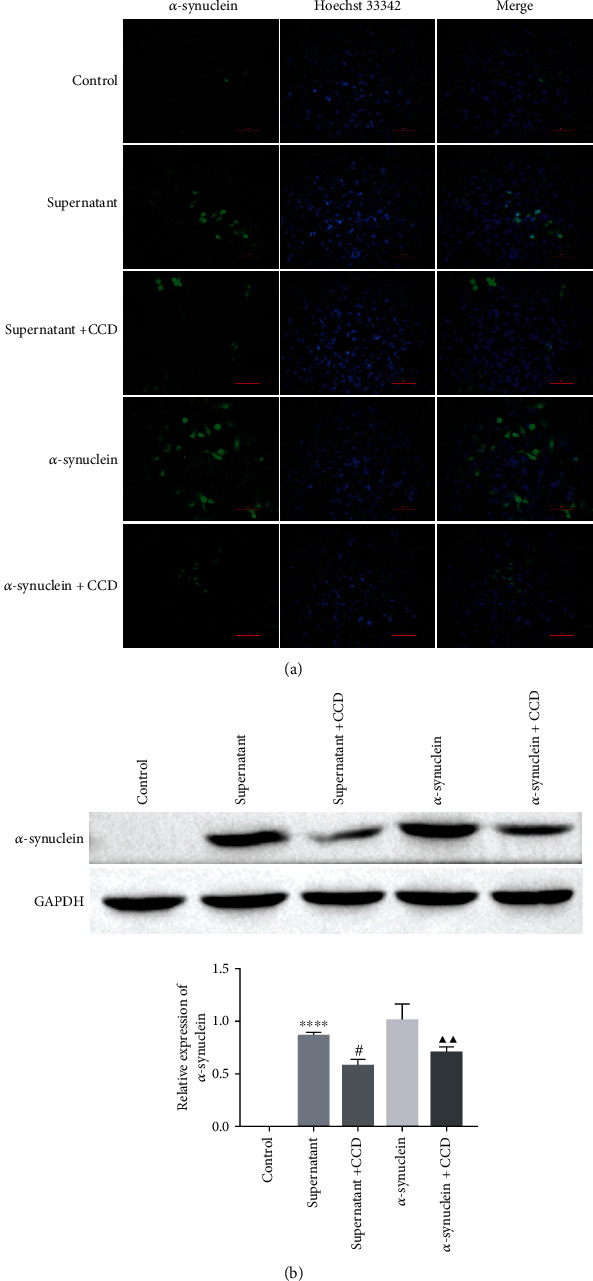
*α*-Synuclein enters microglia via cytophagy. (a) The expression of *α*-synuclein in BV-2 cells was detected by immunofluorescence. Scale bar = 100 px. (b) The expression of *α*-synuclein was detected by western blot, ^∗∗∗∗^*P* < 0.0001 vs. control, ^#^*P* < 0.05 vs. supernatant, and ^▲▲^*P* < 0.01 vs*. α*-synuclein (note: control group: treated small glial cells (BV-2) with PBS; supernatant group: extracted rotenone-treated MN9D cell supernatant cocultured with BV-2 cells; CCD: phagocytosis inhibitor cytochalasin D).

The experimental results obtained from the immunofluorescence experiments showed that the *α*-synuclein fluorescent signal appeared in BV-2 cells in the supernatant group, while the expression of the *α*-synuclein fluorescent signal in BV-2 cells was significantly reduced after the addition of the phagocytosis inhibitor CCD. Similarly, the *α*-synuclein protein solution group showed a stronger *α*-synuclein fluorescent signal, while the addition of the phagocytosis inhibitor CCD also significantly reduced the expression of the *α*-synuclein fluorescent signal in microglia (Figure 3(a)). Moreover, after observing the western blot results, we found that compared with the control group, the rotenone-treated dopaminergic neuronal cell supernatant group and *α*-synuclein protein solution group had upregulated expression of *α*-synuclein, while the addition of CCD decreased the expression of *α*-synuclein (Figure 3(b)). These data suggest that *α*-synuclein can enter microglia through cytophagocytosis.

### 3.4. Effect of *α*-Synuclein on Autophagy and the Inflammatory Response in Microglia

**Figure 4:**
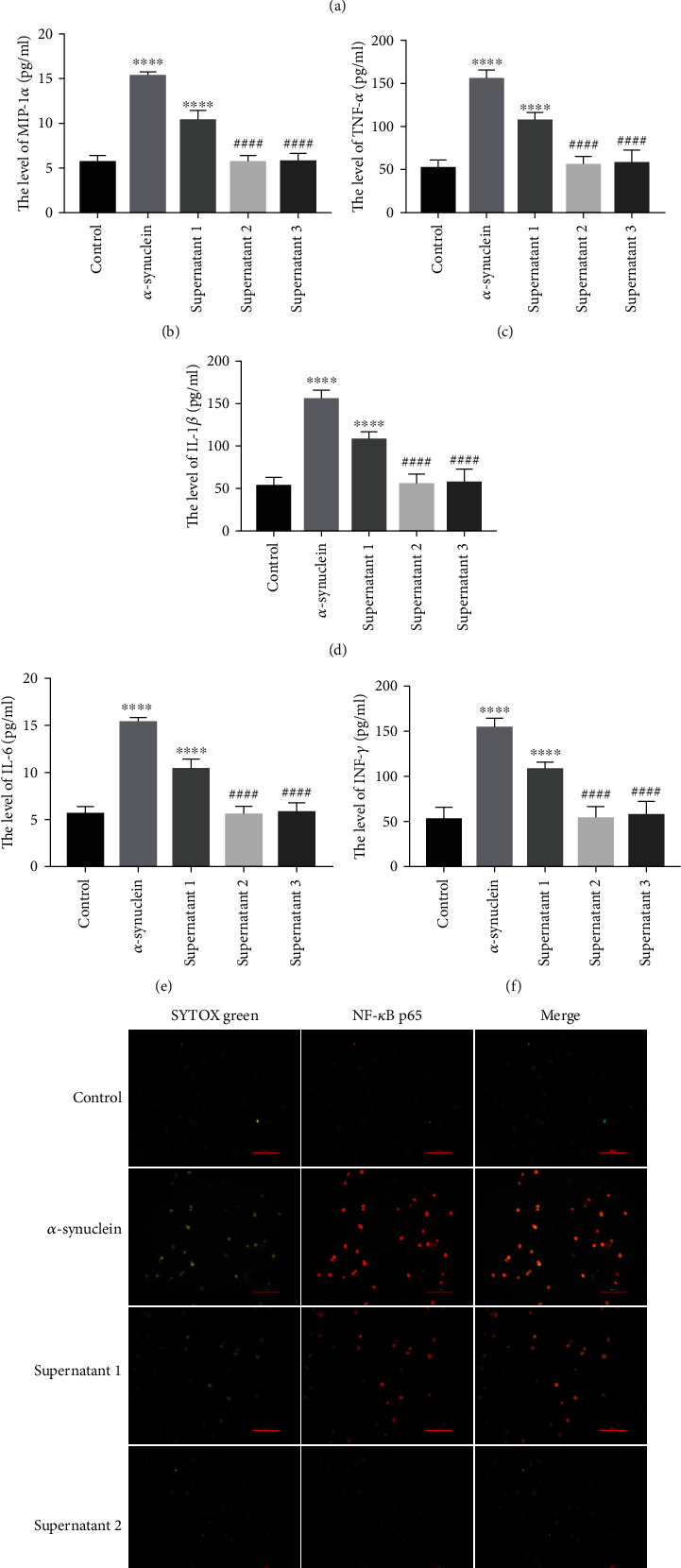
Effect of *α*-synuclein on autophagy and the inflammatory response of microglia. (a) The expression of *α*-synuclein in BV-2 cells was detected by immunofluorescence. Scale bar = 100 px. (b) The level of MIP-1*α* was detected by ELISA, ^∗∗∗∗^*P* < 0.0001 vs. control and ^####^*P* < 0.0001 vs. supernatant 1. (c) The level of TNF-*α* was detected by ELISA, ^∗∗∗∗^*P* < 0.0001 vs. control and ^####^*P* < 0.0001 vs. supernatant 1. (d) The level of IL-6 was detected by ELISA, ^∗∗∗∗^*P* < 0.0001 vs. control and ^####^*P* < 0.0001 vs. supernatant 1. (e) The level of TNF-*α* was detected by ELISA, ^∗∗∗∗^*P* < 0.0001 vs. control and ^####^*P* < 0.0001 vs. supernatant 1. (f) The level of INF-*γ* was detected by ELISA, ^∗∗∗∗^*P* < 0.0001 vs. control and ^####^*P* < 0.0001 vs. supernatant 1. (g) NF-*κ*B p65 nuclear translocation assay, scale bar = 100 px. (h) The expressions of LC3 II/I, Beclin 1, and p62 were detected by western blot, ^∗∗^*P* < 0.01, ^∗∗∗^*P* < 0.001, and ^∗∗∗∗^*P* < 0.0001 vs. control and ^#^*P* < 0.05, ^##^*P* < 0.01, ^###^*P* < 0.001, and ^####^*P* < 0.0001 vs. supernatant 1 (note: supernatant 1 group: the supernatant of MN9D cells overexpressing MALAT1 was cocultured with BV2 cells; supernatant 2 group: the supernatant of MN9D cells cotransfected with pcDNA-MALAT1+miR-23b-3p mimic was cocultured with BV2; supernatant 3 groups: the supernatant of MN9D cells cotransfected with pcDNA-MALAT1+sh-*α*-synuclein was cocultured with BV2).

We added *α*-synuclein to BV2 cells in culture (*α*-synuclein group). The supernatant of MN9D cells overexpressing MALAT1 was cocultured with BV2 cells (supernatant 1 group), and MN9D cells were treated with MALAT1 overexpression and transfected with miR-23b-3p mimic or had *α*-synuclein knocked down. Then, the MN9D cell supernatants were cocultured with BV2 cells, which were recorded as the supernatant 2 group and supernatant 3 group. The effect of *α*-synuclein on autophagy and the inflammatory response of BV2 cells were detected. The expression of *α*-synuclein was detected by immunofluorescence, and phagocytosis of *α*-synuclein by BV-2 cells was observed. The results showed that the control group had no *α*-synuclein fluorescence signal, while the supernatant 1 group (supernatant of MN9D cells overexpressing MALAT1) cocultured with BV2 cells showed a strong *α*-synuclein fluorescence signal compared with the control group. The supernatant 2 and supernatant 3 groups had weak fluorescence signals (Figure 4(a)). ELISA results showed that compared with the control group, the expression of MIP-1*α*, a marker of microglial activation, and the proinflammatory cytokines TNF-*α*, IL-1*β*, IL-6 and INF-*γ* was significantly upregulated in the *α*-synuclein group and supernatant 1 group. Furthermore, compared with the supernatant 1 group, the expression levels of MIP-1*α*, TNF-*α*, IL-1*β*, IL-6, and INF-*γ* in the supernatant 2 and supernatant 3 groups were downregulated (Figures 4(b)–4(f)).

Immunofluorescence experiments were used to observe the nuclear translocation of NF-*κ*B p65. The results showed that compared with the control group, the *α*-synuclein group significantly enhanced the localization of NF-*κ*B p65 in the nucleus of BV-2 cells, and the localization of NF-*κ*B p65 in the nucleus of BV-2 cells in the supernatant 1 group was also significantly increased. However, the localization of NF-*κ*B p65 in the nucleus of BV-2 cells was significantly decreased in the supernatant 2 and supernatant 3 groups compared with the supernatant 1 group (Figure 4(g)). Moreover, after observing the western blot results, we found that the expression of Beclin 1 and LC3 II/I was downregulated, and p62 was upregulated in the *α*-synuclein group and supernatant 1 group. Moreover, the expression of Beclin 1 and LC3 II/I was upregulated, and the expression of p62 was downregulated in the supernatant 2 and supernatant 3 groups compared with the supernatant 1 group (Figure 4(h)). These results suggest that *α*-synuclein affects microglial autophagy and inflammatory responses.

### 3.5. *α*-Synuclein Affects Microglial Activation by Mediating Autophagy

**Figure 5:**
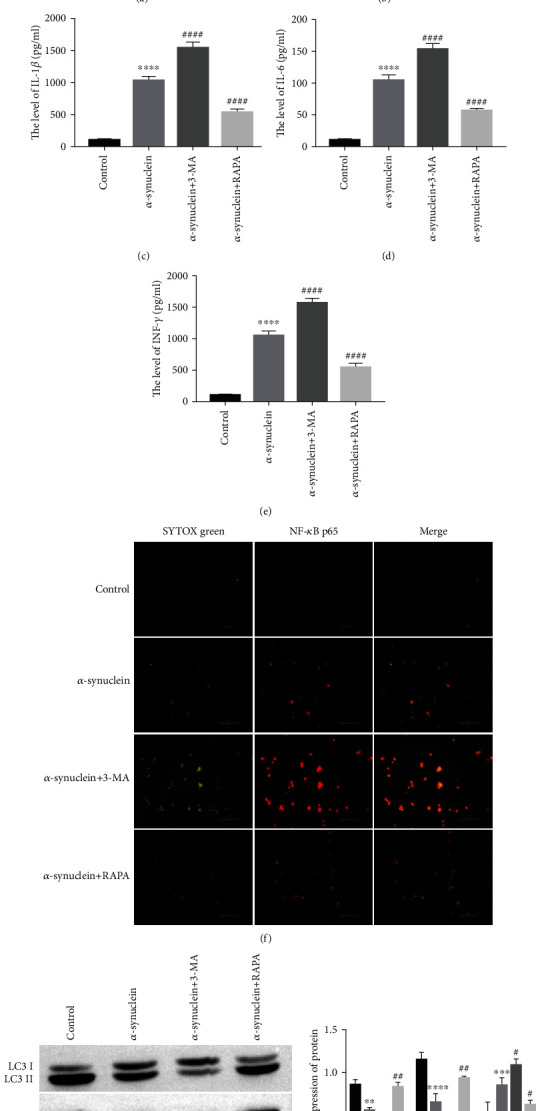
*α*-Synuclein affects microglial activation by mediating autophagy. (a) The level of MIP-1*α* was detected by ELISA, ^∗∗∗∗^*P* < 0.0001 vs. control and ^####^*P* < 0.0001 vs. *α*-synuclein. (b) The level of TNF-*α* was detected by ELISA, ^∗∗∗∗^*P* < 0.0001 vs. control and ^####^*P* < 0.0001 vs. *α*-synuclein. (c) The level of IL-1*β* was detected by ELISA, ^∗∗∗∗^*P* < 0.0001 vs. control and ^####^*P* < 0.0001 vs. *α*-synuclein. (d) The level of IL-6 was detected by ELISA, ^∗∗∗∗^*P* < 0.0001 vs. control and ^####^*P* < 0.0001 vs. *α*-synuclein. (e) The level of INF-*γ* was detected by ELISA, ^∗∗∗∗^*P* < 0.0001 vs. control and ^####^*P* < 0.0001 vs. *α*-synuclein. (f) NF-*κ*B p65 nuclear translocation assay, scale bar = 100 px. (g) The expressions of LC3 II/I, Beclin 1, and p62 were detected by western blot, ^∗∗^*P* < 0.01, ^∗∗∗^*P* < 0.001, and ^∗∗∗∗^*P* < 0.0001 vs. control, ^#^*P* < 0.05, ^##^*P* < 0.01 vs. *α*-synuclein.

To investigate the specific mechanism of activation of microglia by *α*-synuclein, we conducted *α*-synuclein coculture with BV2 cells, followed by treatment with an autophagy inhibitor (3-MA) or autophagy activator (RAPA). The ELISA results showed that compared with the control group, the expression levels of the microglial activation marker MIP-1*α* and the proinflammatory cytokines TNF-*α*, IL-1*β*, IL-6, and INF-*γ* were significantly upregulated in the *α*-synuclein group. 3-MA treatment further increased the expression of MIP-1*α*, TNF-*α*, IL-1*β*, IL-6, and INF-*γ*, while RAPA reversed the promoting effect of *α*-synuclein on the release of inflammatory factors (Figures 5(a)–5(e)).

Immunofluorescence experiments were used to observe the nuclear translocation of NF-*κ*B p65. The results showed that compared with the control group, *α*-synuclein increased the localization of NF-*κ*B p65 in the nucleus of BV-2 cells. Compared with the *α*-synuclein group, the localization of NF-*κ*B p65 in the nucleus of BV-2 cells was further increased in the *α*-synuclein+3-MA group, and the localization of NF-*κ*B p65 in the nucleus of BV-2 cells was decreased in the *α*-synuclein+RAPA group (Figure 5(f)). The western blot results showed that the -autophagy inhibition effect of *α*-synuclein on BV-2 cells could be further enhanced by 3-MA treatment but reversed by RAPA treatment (Figure 5(g)). These results suggest that *α*-synuclein affects the activation of microglia by mediating autophagy.

### 3.6. Activation and Autophagy-Impaired Microglia Promote Apoptosis in Dopaminergic Neuronal Cells

We know that activated microglia release proinflammatory cytokines that can lead to neuronal death. Impaired autophagy of microglia is also associated with neurodegenerative diseases. Both autophagy and activation are regulated by *α*-synuclein, and activated and autophagy-impaired microglia promote apoptosis in dopaminergic neuronal cells. We cocultured the supernatants of *α*-synuclein, *α*-synuclein+3-MA, and *α*-synuclein+RAPA-treated BV-2 cells with MN9D cells.

**Figure 6:**
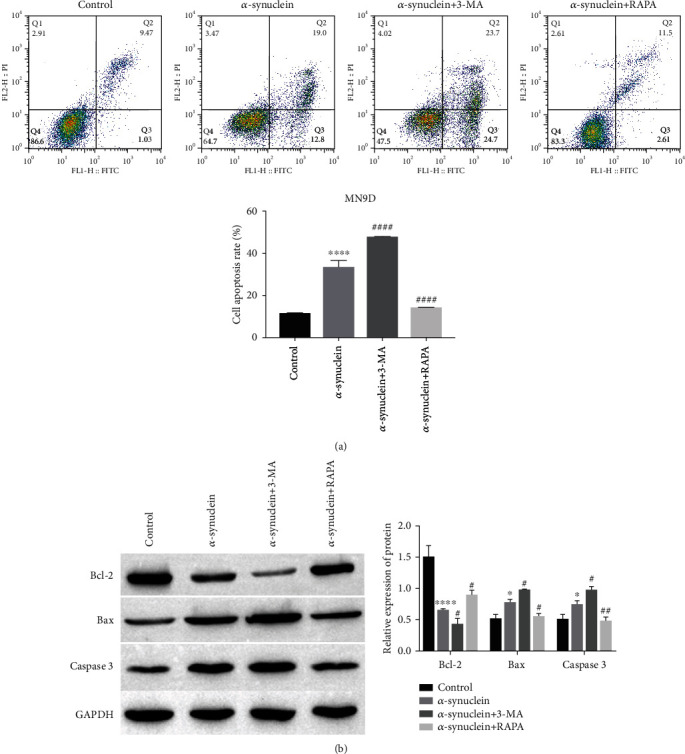
Effect of activation and autophagy-impaired microglia on apoptosis of dopaminergic neuronal cells. (a) Apoptosis of dopaminergic neurons in MN9D cells was detected by flow cytometry, ^∗∗∗∗^*P* < 0.0001 vs. control and ^####^*P* < 0.0001 vs. *α*-synuclein. (b) The expressions of Bcl-2, Bax, and Caspase 3 were detected by western blot, ^∗^*P* < 0.05 and ^∗∗∗∗^*P* < 0.0001 vs. control and ^#^*P* < 0.05 and ^##^*P* < 0.01 vs. *α*-synuclein.

Flow cytometry detection of MN9D cell apoptosis showed that compared with the control group, the apoptosis of MN9D cells in the *α*-synuclein group (supernatant of *α*-synuclein-treated BV-2 cocultured with MN9D) was increased (Figure 6(a)). Compared with the *α*-synuclein group, MN9D cell apoptosis was further increased in the *α*-synuclein+3-MA group (supernatant of *α*-synuclein+3-MA-treated BV-2 cells cocultured with MN9D cells) (Figure 6(a)). The apoptosis of MN9D cells in the *α*-synuclein+RAPA group (supernatant of *α*-synuclein+RAPA-treated BV-2 cells cocultured with MN9D) was decreased compared with the *α*-synuclein group (Figure 6(a)). We also know from the western blot analysis results of apoptosis-related proteins that the expression of Bcl-2 showed a downward trend, and the expression of Bax and Caspase 3 showed an opposite trend in the *α*-synuclein group. Compared with the *α*-synuclein group, the expression of Bcl-2 in the *α*-synuclein+3-MA group was downregulated, the expression of Bax and Caspase 3 was upregulated, the expression of Bcl-2 was upregulated, and the expression of Bax and Caspase 3 was downregulated in the *α*-synuclein+RAPA group (Figure 6(b)).

## 4. Discussion

Parkinson's disease is a common neurodegenerative disease, and most patients are over the age of 60 [44]. The patient's ability to move is severely impaired, and the body and limbs will tremble involuntarily. After suffering from this disease for some time, the patients become unable to engage in self-care and their mental and physical health is greatly affected. At the same time, Parkinson's disease will place considerable economic and mental pressure on the patient's family. Although many people suffer from Parkinson's disease worldwide, there is currently no effective means to prevent or treat the disease. Therefore, it is very meaningful to seek a reliable new drug or medical solution.

Previous studies have shown that PD pathogenesis is associated with MALAT1, miR-23b-3p, and *α*-synuclein. The lncRNA MALAT1 promotes inflammatory vesicle activation through epigenetic suppression of Nrf2 in PD [45]. In PD patients, miR-23b-3p was identified as a PD-associated circulating miRNA [21]. In addition, overexpression of *α*-Syn and miRNA was found in human samples in PD, AD, and dementia. Our results also show that differential expression of MALAT1, miR-23b-3p, and *α*-synuclein is associated with dopaminergic neuronal cell injury and that dopaminergic neuronal cell injury results in the accumulation of large amounts of *α*-synuclein protein. Therefore, the evaluation of these molecules in urine in free form or associated with extracellular vesicles in biological fluids may lead to early biomarkers for clinical diagnosis [20].

We constructed a PD model by damaging dopaminergic neuronal cells with rotenone to investigate the specific mechanism of PD pathogenesis. Rotenone promoted apoptosis of damaged dopaminergic neuronal cells, and dopaminergic neuronal cells accumulated large amounts of *α*-synuclein protein when damaged. Furthermore, our study found that *α*-synuclein caused impaired cellular autophagy, leading to microglial activation and promoting inflammatory responses. Chronic inflammation is part of the pathogenesis of PD, and it was also found that the levels of proinflammatory cytokines, including IL-1*β* and IL-6, in the cerebrospinal fluid of PD patients are markedly increased [46]. Impaired autophagy increases the sensitivity of microglia to *α*-synuclein, which leads to microglial activation to release macrophage inflammatory protein-1*α* (MIP-1*α*) and proinflammatory factors, thereby triggering an inflammatory response that promotes the development of PD [37]. Our study results are consistent with these findings.

However, miR-23b-3p is thought to have a unique function in regulating *α*-synuclein [21]. In this study, the target binding site between miR-23b-3p and *α*-synuclein was predicted by StarBase. Moreover, the binding relationship between MALAT1 and miR-23b-3p was also verified by a dual luciferase gene reporter assay. After summarizing and analyzing the experimental data, we learned from western blot experiments that miR-23b-3p targets and negatively regulates the expression of *α*-synuclein. miR-23b has been previously shown to reduce neuronal apoptosis caused by neuroinflammation, so it is not difficult to hypothesize that it is also inextricably linked to the pathogenesis of PD [47, 48]. Furthermore, a review of the literature revealed that the lncRNA MALAT1 is a clear booster of PD-induced inflammation [45]. MALAT1 exhibits an antagonistic or competitive relationship with miR-23b-3p, and when the former is abundantly expressed, the inhibitory effect of the latter on ATG12 is greatly alleviated, resulting in chemically induced autophagy as well as chemoresistance in GC cells [49]. We found that MALAT1 targets and negatively regulates miR-23b-3p, thereby promoting *α*-synuclein expression, as a way to influence PD. We used the bioinformatics website StarBase, along with dual luciferase gene reporter assays and RT-qPCR assays, to establish this link.

In conclusion, our current findings suggest that MALAT1 is upregulated in PD models and that MALAT1 contributes to the activation of inflammatory vesicles in microglia. The potential mechanism of action is the induction of impaired autophagy and inflammatory responses in microglia through regulation of the miR-23b-3p/*α*-synuclein molecular axis promoting dopaminergic neuronal cell apoptosis with *α*-synuclein nucleoprotein binding to and affecting its endocytosis and intercellular transmission. These studies suggest that lncRNA MALAT1 may be a new research entry point for clinical application in Parkinson's disease due to its effects on dopaminergic neuron apoptosis.

In conclusion, our findings suggest that MALAT1 acts as a miR-23b-3p sponge, regulating *α*-synuclein expression to induce microglial autophagy impairment and inflammatory responses to promote dopaminergic neuronal apoptosis through cellular transmission. This provides a new potential therapeutic target for PD.

## Figures and Tables

**Table 1:** RT-qPCR primer sequences.

Target	Sequence (F: forward primer; R: reverse primer)
miR-23b-3p	F: 5′-CGATCACATTGCCAGGGAT-3′
	R: 5′-AGTGCAGGGTCCGAGGTATT-3′

MALAT1	F: 5′-AAAGCAAGGTCTCCCCACAAG-3′
	R: 5′-GGTCTGTGCTAGATCAAAAGGCA-3′

U6	F: 5′-CTCGCTTCGGCAGCACA-3′
	R: 5′-AACGCTTCACGAATTTGCGT-3′

## Data Availability

The datasets used and/or analyzed during the current study are available from the corresponding authors upon reasonable request.

## References

[1] Pringsheim T., Jette N., Frolkis A., Steeves T. D. (2014). The prevalence of Parkinson’s disease: a systematic review and meta-analysis. *Movement Disorders*.

[2] Drui G., Carnicella S., Carcenac C. (2014). Loss of dopaminergic nigrostriatal neurons accounts for the motivational and affective deficits in Parkinson’s disease. *Molecular Psychiatry*.

[3] Nussbaum R. L. (2017). The identification of alpha-synuclein as the first Parkinson disease gene. *Journal of Parkinson's Disease*.

[4] Kim Y. S., Joh T. H. (2006). Microglia, major player in the brain inflammation: their roles in the pathogenesis of Parkinson’s disease. *Experimental & Molecular Medicine*.

[5] Lenz K. M., Nelson L. H. (2018). Microglia and beyond: innate immune cells as regulators of brain development and behavioral function. *Frontiers in Immunology*.

[6] Smith J. A., Das A., Ray S. K., Banik N. L. (2012). Role of pro-inflammatory cytokines released from microglia in neurodegenerative diseases. *Brain Research Bulletin*.

[7] Stephenson J., Nutma E., van der Valk P., Amor S. (2018). Inflammation in CNS neurodegenerative diseases. *Immunology*.

[8] Harries L. W. (2012). Long non-coding RNAs and human disease. *Biochemical Society Transactions*.

[9] Qureshi I. A., Mehler M. F. (2013). Long non-coding RNAs: novel targets for nervous system disease diagnosis and therapy. *Neurotherapeutics*.

[10] Chen R., Liu Y., Zhuang H. (2017). Quantitative proteomics reveals that long non-coding RNA MALAT1 interacts with DBC1 to regulate p 53 acetylation. *Nucleic Acids Research*.

[11] Kraus T. F. J., Haider M., Spanner J., Steinmaurer M., Dietinger V., Kretzschmar H. A. (2017). Altered long noncoding RNA expression precedes the course of Parkinson’s disease-a preliminary report. *Molecular Neurobiology*.

[12] Gutschner T., Hämmerle M., Eißmann M. (2013). The noncoding RNAMALAT1Is a critical regulator of the metastasis phenotype of lung cancer cells. *Cancer Research*.

[13] Bernard D., Prasanth K. V., Tripathi V. (2010). A long nuclear-retained non-coding RNA regulates synaptogenesis by modulating gene expression. *The EMBO Journal*.

[14] Lipovich L., Dachet F., Cai J. (2012). Activity-dependent human brain coding/noncoding gene regulatory networks. *Genetics*.

[15] Zhang Q. S., Wang Z. H., Zhang J. L., Duan Y. L., Li G. F., Zheng D. L. (2016). Beta-asarone protects against MPTP-induced Parkinson’s disease via regulating long non-coding RNA MALAT1 and inhibiting *α*-synuclein protein expression. *Biomedicine & Pharmacotherapy*.

[16] Bartel D. P. (2004). MicroRNAs: genomics, biogenesis, mechanism, and function. *Cell*.

[17] Khoo S. K., Petillo D., Kang U. J. (2012). Plasma-based circulating microRNA biomarkers for Parkinson’s disease. *Journal of Parkinson's Disease*.

[18] Cardo L. F., Coto E., Mena L. (2013). Profile of microRNAs in the plasma of Parkinson’s disease patients and healthy controls. *Journal of Neurology*.

[19] Yao Y. F., Qu M. W., Li G. C., Zhang F. B., Rui H. C. (2018). Circulating exosomal miRNAs as diagnostic biomarkers in Parkinson’s disease. *European Review for Medical and Pharmacological Sciences*.

[20] Giri B., Seamon M., Banerjee A. (2022). Emerging urinary alpha-synuclein and miRNA biomarkers in Parkinson’s disease. *Metabolic Brain Disease*.

[21] Cai M., Chai S., Xiong T. (2021). Aberrant expression of circulating microRNA leads to the dysregulation of alpha-synuclein and other pathogenic genes in Parkinson’s disease. *Frontiers in Cell and Development Biology*.

[22] Braak H., Del Tredici K., Rub U., de Vos R. A., Jansen Steur E. N., Braak E. (2003). Staging of brain pathology related to sporadic Parkinson’s disease. *Neurobiology of Aging*.

[23] Desplats P., Lee H. J., Bae E. J. (2009). Inclusion formation and neuronal cell death through neuron-to-neuron transmission of alpha-synuclein. *Proceedings of the National Academy of Sciences of the United States of America*.

[24] Zhang W., Wang T., Pei Z. (2005). Aggregated *α*-synuclein activates microglia: a process leading to disease progression in Parkinson’s disease. *The FASEB Journal*.

[25] Liang Y., Zhou T., Chen Y. (2017). Rifampicin inhibits rotenone-induced microglial inflammation via enhancement of autophagy. *Neurotoxicology*.

[26] Das G., Shravage B. V., Baehrecke E. H. (2012). Regulation and Function of Autophagy during Cell Survival and Cell Death. *Cold Spring Harbor Perspectives in Biology*.

[27] Wilson C. M., Magnaudeix A., Yardin C., Terro F. (2014). Autophagy dysfunction and its link to Alzheimer’s disease and type II diabetes mellitus. *CNS & Neurological Disorders Drug Targets*.

[28] Li L., Zhang X., Le W. (2010). Autophagy dysfunction in Alzheimer’s disease. *Neurodegenerative Diseases*.

[29] Wang F., Jia J., Rodrigues B. (2017). Autophagy, metabolic disease, and pathogenesis of heart dysfunction. *The Canadian Journal of Cardiology*.

[30] Yang D. J., Zhu L., Ren J., Ma R. J., Zhu H., Xu J. (2015). Dysfunction of autophagy as the pathological mechanism of motor neuron disease based on a patient-specific disease model. *Neuroscience Bulletin*.

[31] El-Khider F., McDonald C. (2016). Links of autophagy dysfunction to inflammatory bowel disease onset. *Digestive Diseases*.

[32] Cortes C. J., La Spada A. R. (2014). The many faces of autophagy dysfunction in Huntington’s disease: from mechanism to therapy. *Drug Discovery Today*.

[33] Narendra D., Tanaka A., Suen D. F., Youle R. J. (2008). Parkin is recruited selectively to impaired mitochondria and promotes their autophagy. *The Journal of Cell Biology*.

[34] Alvarez-Erviti L., Rodriguez-Oroz M. C., Cooper J. M. (2010). Chaperone-mediated autophagy markers in Parkinson disease brains. *Archives of Neurology*.

[35] Dehay B., Bové J., Rodríguez-Muela N. (2010). Pathogenic lysosomal depletion in Parkinson’s disease. *The Journal of Neuroscience*.

[36] Vives-Bauza C., Zhou C., Huang Y. (2010). PINK1-dependent recruitment of Parkin to mitochondria in mitophagy. *Proceedings of the National Academy of Sciences of the United States of America*.

[37] Cheng J., Liao Y., Dong Y. (2020). Microglial autophagy defect causes Parkinson disease-like symptoms by accelerating inflammasome activation in mice. *Autophagy*.

[38] Komatsu M., Waguri S., Chiba T. (2006). Loss of autophagy in the central nervous system causes neurodegeneration in mice. *Nature*.

[39] Hara T., Nakamura K., Matsui M. (2006). Suppression of basal autophagy in neural cells causes neurodegenerative disease in mice. *Nature*.

[40] Friedman L. G., Lachenmayer M. L., Wang J. (2012). Disrupted autophagy leads to dopaminergic axon and dendrite degeneration and promotes presynaptic accumulation of *α*-synuclein and LRRK2 in the brain. *The Journal of Neuroscience*.

[41] Nixon R. A. (2013). The role of autophagy in neurodegenerative disease. *Nature Medicine*.

[42] Yao L., Zhu Z., Wu J. (2019). MicroRNA-124 regulates the expression of p62/p38 and promotes autophagy in the inflammatory pathogenesis of Parkinson’s disease. *The FASEB Journal*.

[43] Zhang L. M., Wang M. H., Yang H. C. (2019). Dopaminergic neuron injury in Parkinson’s disease is mitigated by interfering lncRNA SNHG14 expression to regulate the miR-133b/ *α*-synuclein pathway. *Aging (Albany NY)*.

[44] Elbaz A., Carcaillon L., Kab S., Moisan F. (2016). Epidemiology of Parkinson’s disease. *Revue Neurologique (Paris)*.

[45] Cai L. J., Tu L., Huang X. M. (2020). lncRNA MALAT1 facilitates inflammasome activation via epigenetic suppression of Nrf2 in Parkinson’s disease. *Molecular Brain*.

[46] Lema Tome C. M., Tyson T., Rey N. L., Grathwohl S., Britschgi M., Brundin P. (2013). Inflammation and *α*-synuclein’s prion-like behavior in Parkinson’s disease--is there a link?. *Molecular Neurobiology*.

[47] Chen Q., Xu J., Li L. (2014). MicroRNA-23a/b and microRNA-27a/b suppress Apaf-1 protein and alleviate hypoxia-induced neuronal apoptosis. *Cell Death & Disease*.

[48] Hu L., Zhang H., Wang B., Ao Q., Shi J., He Z. (2019). MicroRNA-23b alleviates neuroinflammation and brain injury in intracerebral hemorrhage by targeting inositol polyphosphate multikinase. *International Immunopharmacology*.

[49] YiRen H., Ying Cong Y., Sunwu Y., Keqin L., Xiaochun T., Senrui C. (2017). Long noncoding RNA MALAT1 regulates autophagy associated chemoresistance via miR-23b-3p sequestration in gastric cancer. *Molecular Cancer*.

